# Children with sickle cell disease: are they protected from serious COVID-19?

**DOI:** 10.3389/fped.2024.1337377

**Published:** 2024-10-07

**Authors:** Walaa Aboulkasem Shahin, Hayam Aldeeb, Majed Alsulami, Abdullah Tammas, Fatma Albatniji, Aljawhara Almanea, Abdalla Mohamed Zayed, Fahad Alabbas, Azzah Alzahrani, Tahani Bin Ali, Ghaleb Elyamany, Rana Hassan Almaghrabi, Huda Elfaraidi

**Affiliations:** ^1^ Department of Pediatrics, Specialized Children's Hospital, Cairo University, Cairo, Egypt; ^2^ Department of Pediatrics, Prince Sultan Military Medical City, Riyadh, Saudi Arabia; ^3^ Hematology Division, Department of Pediatrics, King Fahad Armed Forces Hospital, Jeddah, Saudi Arabia; ^4^ Department of Pediatrics, King Fahad Medical City, Riyadh, Saudi Arabia; ^5^ Department of Central Military Laboratory and Blood Bank, Prince Sultan Military Medical City, Riyadh, Saudi Arabia

**Keywords:** COVID-19, pediatrics, sickle cell, hydroxyurea, Saudi Arabia

## Abstract

**Background:**

COVID-19, the pandemic that hit the world in 2020, resulted in millions of deaths, with the elderly and adults succumbing to the disease more often than children. However, the presence of underlying morbidities increased the risk of death. Sickle cell disease (SCD) was previously classified as a major risk factor for severe COVID-19 disease. However, presently, there are only a limited number of studies that identify the clinical course of children with SCD and COVID-19.

**Methods:**

We conducted a retrospective observational study on children with SCD admitted due to COVID-19 at three different institutions in Saudi Arabia between March 2020 and March 2022. We studied the demographic and clinical characteristics of patients admitted to the hospital.

**Results:**

Seventy-six patients with SCD had PCR-confirmed SARS-CoV-2 during the study period; 50.0% of our patient population were children (6–12 years old). Gender was evenly distributed, with 53.9% girls and 46.1% boys. Symptoms more commonly related to the COVID-19 infection included fever, cough, malaise, and vomiting. Chest x-ray findings revealed mild and non-specific symptoms only in approximately one-third (28) of the included children. The most common symptoms associated with SCD were vaso-occlusive crisis (47.4%) and abdominal pain (11.8%). The overall general appearance of most of the patients was reassuring. The median length of hospital stay was 4.2 ± 2.7 days. The mean white blood cell count was 11.4 ± 5.2 × 10^9^/L, and the mean hemoglobin level was 8.3 ± 1.5 g/dl. Despite the fact that higher levels of mean D-dimer, lactate dehydrogenase, and ferritin were reported in these patients, the clinical outcome was not affected. All recruited patients received hydroxyurea as maintenance therapy. The outcome of our study was reassuring, with no significant morbidity or mortality observed among the recruited patients.

**Conclusion:**

Despite SCD being a chronic disease with known specific complications, there has been a claim that COVID-19 infection adds further risk. The results of this study suggest that the overall outcome of COVID-19 was favorable, with no reported mortality. Further research is needed to understand the factors that contributed to this favorable outcome. In children with SCD, it is still questionable whether hydroxyurea is one of the protective factors against severe COVID-19. Validation through large-scale research is recommended.

## Introduction

The World Health Organization classified COVID-19 (SARS-CoV-2), which resulted in more than 500 million confirmed illnesses and more than 6 million fatalities around the world, as a global pandemic in January 2020 (1). The reported high morbidity and death rates were more common in the elderly and individuals with underlying chronic illnesses, such as diabetes, obesity, and SCD, during the early period of the pandemic (2). Most children had no symptoms or only minor signs. Underlying conditions were more common among school-aged children with severe outcomes related to COVID-19: among school-aged children who were hospitalized, admitted to an intensive care unit (ICU), or who died, 23%, 38%, and 33%, respectively, had at least one underlying condition (3). A multicenter investigation involving 2,293 hospitalized children infected with the coronavirus showed chronic pulmonary disease and neurologic abnormalities as the most significant risk factors for severe COVID-19 infection (4). Similar findings were shown in the study done at Children's National Hospital in Washington, DC, which investigated 177 children and adolescents who tested positive for SARS-CoV-2 and had an underlying medical condition. The study verified the association of comorbidities and increased hospitalization rates (5).

Sickle cell disease (SCD) has been described as a significant risk factor for severe COVID-19. The rate of patients with SCD diagnosed with COVID-19 visiting the emergency department, followed by hospitalization, is higher than that of the general population, with vaso-occlusive crisis and acute chest syndrome being the main clinical presentations. Case reports, multicenter studies, and single-center studies have all significantly contributed to the development of knowledge about the impact of COVID-19 in the context of SCDs (6–8). A recent meta-analysis comparing COVID-19 outcomes in individuals with sickle cell disease or trait with the general population suggested that patients with SCD or sickle cell trait (SCT) and SARS-CoV-2 infection present with increased mortality rates compared with the general population (9). Sickle cell disease, an autosomal recessive condition, is prevalent in Saudi Arabia, the Mediterranean region, and sub-Saharan Africa. The results of a nation-wide community-based survey done in Saudi Arabia showed a high prevalence of SCD in the community, where it was detected in 108 of 45,682 children and adolescents, with a prevalence of 24 per 10,000 (10, 11).

On the other hand, some studies suggested that most children with SCD and COVID-19 have mild disease and a minimal risk of death, while some children with SCD are more likely to be hospitalized and require enhanced care than those without SCD. However, children with SCD are less likely to experience COVID-19-related severe illness and death compared with adults with or without SCD. SCD-directed therapies such as transfusion and hydroxyurea may be associated with better COVID-19 outcomes (12). The complications of SCD and the added risk of COVID-19 infection arise when the red blood cells (RBCs), holding either Hemoglobin S (HbS) or a combination of HbS and other variant alleles, meet a low-oxygen environment. Consequently, these cells undergo polymerization and stiffening and become susceptible to hemolysis. The altered blood flow contributes to the onset of symptoms such as acute chest syndrome (13, 14).

According to a report published by the SECURE registry on 750 cases of SCD with COVID-19, there was a rise in morbidity and mortality rates in this affected population compared with the general population, wherein 60% of adults and 40% of children were admitted to the hospital, and 5.8% and 8.8% were admitted to the ICU (7). In contrast, an earlier study done in Saudi Arabia in 2021 on children with SCD and COVID-19 showed favorable outcomes and a good prognosis, in which all patients recovered and were discharged (15, 16).

In this study, our aim was to assess the impact of COVID-19 on pediatric patients with SCD using data from three big centers in the Kingdom of Saudi Arabia. We recruited only those children admitted due to COVID-19 and studied their clinical manifestations and laboratory data.

## Methods

### Study population

All pediatric patients (aged 1–14 years) diagnosed with SCD and confirmed positive for SARS-CoV-2 through real-time polymerase chain reaction (RT-PCR) were included in this study if they were hospitalized between March 2020 and March 2022.

With regard to the long-term management of children with SCD, we follow the recommendations outlined in the National Heart, Lung, and Blood Institute Expert Panel Report published in 2014. According to this report, hydroxyurea therapy at a dosage of 20 mg/kg/day is recommended for all children with sickle cell disease aged nine months and older (17). The starting dose was 15 mg/kg/day for all patients, which was titrated according to the patient's tolerance and then maintained at an average of 20 mg/kg/day. The maximum therapeutic dose (MTD) differs from 15 to 30 mg/kg/day depending on the tolerance and physician assessment for dose increment. By the time of enrollment, all included children had been on a maintenance average dose of hydroxyurea of 20 mg/kg/day.

We collected data from three medical centers in Saudi Arabia: Prince Sultan Military Medical City (PSMMC) in the central province, King Fahad Armed Forces Hospital (KHAFH) in the western province, and King Fahad Military Medical Complex (KFMMC) Dhahran in the eastern province.

Data were collected retrospectively from the files of patients, including information on demographics, clinical and laboratory characteristics, management, and outcome. In 2020, all included centers implemented standardized COVID-19 management and treatment guidelines in accordance with the recommendations of the Saudi Ministry of Health. We ensured that the confidentiality of all participants whose data were used in the study was maintained. The Ethical Committee of Prince Sultan Military Medical City (PSMMC) approved the study with IRB number (2021-44).

### Statistical analysis

We simultaneously entered the data into a new and updated Microsoft Excel sheet. The data were analyzed using SPSS software version 22.0. Descriptive analysis included the calculation of frequencies and percentages. We represented categorical variables as numbers and described quantitative variables using the mean and SD.

## Results

### Demographics and clinical characteristics

Seventy-six patients with SCD had PCR-confirmed SARS-CoV-2 during the study period, of which 50.0% (*N* = 38) of the patient population were children aged 6–12 years old. Gender was equally matched, with 53.9% (*N* = 41) being girls. At the time of our study, none of the recruited patients had received the COVID-19 vaccine, as it had not yet been approved for use in children. The vaccine had been introduced at the beginning of 2022, after the conclusion of our study. Notably, the last patient recruited in our study was admitted on 30 January 2022 prior to the initiation of mass vaccination of children in the country (18).

The most common symptoms related to COVID-19 were fever, cough, malaise, and vomiting. Abnormalities in the chest x-rays included non-specific findings such as mild atelectasis, patchy infiltration, and peribronchial thickening.

The most common symptoms related to SCD were vaso-occlusive crisis, abdominal crisis, and acute chest syndrome. The general condition of most of our patients was good. The median length of hospitalization was 4.2 ± 2.7 days. All patients recovered without any known complications or mortality. Demographics and clinical characteristics are summarized in Table 1.

**Table 1:** Demographics and baseline clinical characteristics of SCD patients infected with COVID-19.

Characteristics (*N* = 76)	Result
Age (years)	
1–3	10 (13.2%)
3–6	14 (18.4%)
6–12	38 (50.0%)
12–14	14 (18.4%)
Gender	
Female	35 (46.1%)
Male	41 (53.9%)
On Hydroxyurea	76 (100%)
Covid-19 vaccine	Zero
Comorbidities	
Asthma	5 (6.6%)
Stroke (chronic transfusion)	2 (2.6%)
G-6-PD^a^	2 (2.6%)
Brain dysgenesis	1 (1.3%)
Symptoms related to COVID-19	
Fever	64 (84.2%)
Cough	42 (55.3%)
Hypoxia	6 (7.9%)
Vomiting	16 (21.1%)
Diarrhea	9 (11.8%)
Dehydration	14 (18.4%)
Malaise	12 (15.8)
Symptoms related to SCD	
No crisis	18 (23.6%)
Vaso-occlusive crisis	36 (47.4%)
Abdominal crisis	9 (11.8%)
Stroke	2 (2.6%)
Dactylitis	1 (1.3%)
• Acute chest syndrome	10 (13.2%)
General condition	
Healthy looking	64 (84.2%)
Ill-looking^b^	12 (15.8%)
Chest x—ray	
Normal	48 (63.2%)
Abnormal^b^	28 (36.8%)
Days of admission	4.2 ± 2.7
Recovery	76 (100%)

ICU, intensive care unit; SCD, sickle cell disease.

Data are represented as numbers and percentages or as mean ± SD.

^a^ G-6-P-D, glucose 6 phosphate dehydrogenase deficiency.

^b^ Abnormal x—ray findings include mild atelectasis, patchy infiltration, and peribronchial thickening. Ill-looking children who have fatigue, decreased activity, or decreased appetite.

The demographics of the studied population showed that approximately half of the patients were 6–12 years of age. The most common presenting symptoms were fever and cough, and the less common ones were vomiting and diarrhea. The most common presenting symptom related to SCD is vaso-occlusive crisis, with acute chest syndrome and stroke being less related to it. Twenty five percent of the patients only had symptoms related to COVID-19 with no sickle cell disease–related crisis. All patients, even those with comorbidities, showed full recovery. Chest x-ray findings revealed non-specific and mild symptoms such as peribronchial thickening and mild atelectasis, which were noticed in 37 percent of patients.

Laboratory findings of the studied population are presented in Table 2. The hemoglobin levels of the patients showed a slight drop below the normal range for SCD, which is between 9 and 10 mg/dl. An elevated median ferritin level of 840.6 ± 698.0 ng/dl was found in a subset of SCD patients with COVID-19 (not routinely requested for all patients).

**Table 2:** Laboratory parameters of SCD patients.

Parameter	Result (mean)	Normal values
WBC (10^9^/L)	11.4 ± 5.2	5–20
Eosinophils	0.2 ± 0.2	0.2–0.9
Lymphocytes	4.1 ± 3.2	2–17
Neutrophils	5.9 ± 3.7	1–9.5
HB (g/dl)	8.3 ± 1.5	12.5–20.5
Platelets (10^9^/L)	350.2 ± 172.1	150–450
D-dimer (μg/ml)	4.5 ± 5.0	0–0.5
Procalcitonin (ng/ml)	0.5 ± 0.9	0–0.05
Ferritin (ng/ml)	840.6 ± 698.0	14–124
LDH (U/L)	525.2 ± 144.7	120–300
Blood culture		
Negative	73 (96.1%)	
Positive	3 (3. 9%)	

HBEP, hemoglobin electrophoresis; WBCs, white blood cells; HB, hemoglobin; LDH, lactate dehydrogenase.

Data are represented as mean ± SD or as numbers and percentages.

Table 3 details other laboratory parameters that were studied only for some patients, with such a study depending on the admitting team. The results showed slight abnormalities in the coagulation profile, namely, the prothrombin time (61.8%, *n* = 47). The rise in the liver function profile was due to a high level of aspartate transferase, (56.6%, *n* = 43). Fifty-one (67.1%) patients had higher levels of C-reactive protein (CRP). Despite high D-dimer, ferritin levels, and lactate dehydrogenase (LDH) being reported in the patients, there were no reported cases of thrombosis or stroke.

**Table 3:** Liver and kidney function in SCD patients.

Parameter	Normal	High
Coagulation profile		
PT	29 (38.2%)	47 (61.8%)
PTT	53 (69.7%)	23 (30.3%)
Fibrinogen	60 (78.9%)	16 (21.1%)
Liver function profile		
AST	33 (43.4%)	43 (56.6%)
ALT	67 (88.2%)	9 (11.8%)
GGT	72 (94.7%)	4 (5.3%)
Kidney function	65 (85.5%)	11 (14.5%)
CRP	25 (32.9%)	51 (67.1%)

PT, prothrombin time; PTT, partial thromboplastin time; ALT, alanine transaminase; AST, aspartate aminotransferase; GGT, gamma-glutamyl transferase; CRP, C-reactive protein.

Data are represented as numbers and percentages.

Half of the SCD patients with COVID-19 needed a blood transfusion. In addition, only 11.8% of patients needed a double volume exchange transfusion (DVET). Furthermore, most patients did not need oxygen therapy, as shown in Table 4.

**Table 4:** The clinical intervention required.

Procedure	Number	Percentage
Blood transfusion		
Yes	38	50.0
No	38	50.0
DVET		
Yes	9	11.8
No	67	88.2
Oxygen therapy		
Yes	6	7.9
No	70	92.1

DVET, double volume exchange transfusion.

Data are represented as numbers and percentages.

## Discussion

Not enough data are available in the literature about COVID-19 presentation in children with SCD. The present study documented the clinical progression of the infection in this population, with variable morbidity and no mortality seen. This result was consistent with that of the earlier smaller-scale study by Kashari et al. in 2021 in Saudi Arabia that shed light on the favorable outcome of SCD with COVID-19 (15).

COVID-19 in children in general manifested in a milder disease form with minimal morbidity and mortality compared with the adult population, as described by multiple studies in the country or worldwide (16, 19).

However, with the presence of an underlying chronic disease, there were expectations that the infection would carry more risk for vulnerable groups. Interestingly, the data available from our study or earlier ones are reassuring to us, yet we must be careful with dealing with fragile patients, by providing them timely and appropriate treatment to protect them from infection.

The French experience during the pandemic concludes that COVID-19, even if potentially severe, does not seem to carry an increased risk of morbidity or mortality in patients with SCD. Most of the patients studied in that country have an SS/SB phenotype and are younger than 45 years. Another study by Panepinto et al. in 2020 stressed that the specific socioeconomic and healthcare access challenges that many people with SCD face (i.e., social determinants of health) need to be considered while implementing prevention measures, as well as a consideration of the challenge of access to early medical care, which might have a good impact on the outcome. Our findings align closely with the results of the SECURE-SCD registry, which was established to gather and archive data concerning patients with SCD and affected by COVID-19 (6, 20).

Our study reported that the most common symptoms related to SCD were vaso-occlusive crisis (47.4%), abdominal crisis (11.8%), and acute chest syndrome (13.2%). These results agree with those reported by Martin et al., who found that the most frequent SCD-specific presenting symptom was vaso-occlusive pain crisis (43%), followed by acute chest syndrome (35%). In addition, 24% of SCD patients with COVID-19 were asymptomatic, 54% had mild illness, 13% had moderate illness, and 9% had severe illness, with no mortality reported (21). Our study showed that 100% of patients recovered without mortality, and 84.2% of patients had a good general condition. Moreover, the mean length of hospitalization was 4.2 ± 2.7 days, and the overall prognosis was favorable.

It is worth noting that all the enrolled children received regular treatment with hydroxyurea, which played a pivotal role in disease control. Fetal hemoglobin (HbF) induction is the most powerful effect of hydroxyurea and provides a direct benefit for people who have SCD. In addition to this, hydroxyurea lowers the number of circulating leukocytes and reticulocytes and alters the expression of adhesion molecules, all of which contributes to vaso occlusion. Furthermore, it is believed to contribute to a reduction in inflammatory mediators and plays a crucial role in nitric oxide synthesis and regulation. This, in turn, can enhance microvasculature in susceptible individuals and potentially improve the prognosis of the disease (22–24).

In addition, given the absence of COVID-19 vaccination at the time of enrollment of our patients and the potential beneficial effects of hydroxyurea, the possibility of the protective role of this therapy against COVID-19 increases.

With regard to the D-dimer finding, it was challenging to interpret the test results due to the D-dimer fluctuations associated with the sickling of RBCs. The D-dimer test is, by itself, used as a risk factor for death in both SCD and normal patients with COVID-19. However, higher serum levels of D-dimers in our studied population did not affect the outcome, as all patients recovered (25–27).

Our results also aligned with a study conducted in Canada involving 185 patients with SCD who were infected with COVID-19. The Canadian study concluded that, despite the higher risk of severe disease in the SCD population compared with the general population, the outcomes were favorable, with no reported deaths. They hypothesized that higher HbF levels may prevent COVID-19-related variants of concern (VOCs). Among hospitalized patients, a non-statistical tendency toward lower HbF was observed in patients who had COVID-19-related acute coronary syndrome (ACS) compared with those without ACS. Higher HbF levels may prevent COVID-19-related ACS (28). This observation was consistent with our current knowledge about HbF. Higher levels of HbF (at baseline or due to hydroxyurea) reduce the polymerization of deoxy sickle hemoglobin and decrease the risks of VOC and ACS (29).

### Study limitations

Retrospective observational nature of our study imposed restrictions on how the data could be analyzed, interpreted, or utilized. Second, the patient sample size was small. Another limitation was the lack of correlation between the HbF levels and the severity of the infection. By considering children with COVID-19, we referred to the literature to compare the clinical outcome of these children with that of children with SCD.

## Conclusion

The insights gained from this study will significantly contribute to the expanding body of research on pediatric SCD and COVID-19. While morbidity in our cohort was variable, we observed no fatalities in children with SCD and COVID-19 infections. Larger studies and longitudinal analyses could offer valuable insights into the potential protective effects of hydroxyurea and its impact on COVID-19 severity and outcomes in this population.

Implementing new guidelines would help lower the unnecessary burden of hospital admissions. In addition, we recommend further research efforts to focus on investigating the emergence of long-term effects of COVID-19 in patients with SCD, including potential complications, sequelae, and impact on disease progression and overall health.

## Data Availability

The raw data supporting the conclusions of this article will be made available by the authors without undue reservation.
